# Rupture de la rate au cours de l'accouchement: à propos d'un cas

**DOI:** 10.4314/pamj.v9i1.71196

**Published:** 2011-06-23

**Authors:** Houda Elbahraoui, Hanane Bouziane, Jamila Akrouch, Amina Lakhdar, Driss Ferhati

**Affiliations:** 1 Service de gynecologie-obstetrique, CHU Ibn Sina Rabat, Maroc

**Keywords:** Rupture, rate, accouchement dystocique

## Abstract

La rupture de rate est une extrême urgence chirurgicale abdominale qui met très rapidement en jeu le pronostic vital. Les étiologies fréquemment retrouvées sont les traumatismes abdominaux par contusions ou plaies pénétrantes. La survenue d'une rupture de rate au cours de l'accouchement est un événement rare. Nous rapportons dans cette observation le cas d'une rupture splénique suite à des expressions abdominales survenue au cours d'un accouchement dystocique.

## Introduction

La survenue d'une rupture de la rate au cours de l'accouchement est un événement très rare et représente une extrême urgence chirurgicale abdominale qui met très rapidement en jeu le pronostic vital maternel. En dehors de l'accouchement la rupture splénique est souvent post-traumatique suite à une contusion ou plaies pénétrantes. Nous allons rapporter l'observation d'une parturiente dont l'accouchement dystocique s'est compliqué d'une rupture de la rate avec issue favorable malgré la gravité du tableau clinique [1].

## Patient et observation

Madame H.A est une patiente de 32 ans, primipare, primigeste, sans antécédents particuliers. Elle a été admise aux urgences de gynécologie-obstétrique du centre hospitalier universitaire de Rabat pour un état de choc après un accouchement dystocique dans une maternité périphérique. Des expressions abdominales ainsi qu'une extraction instrumentale ont été réalisée donnant naissance à un nouveau-né pesant 3050g et dont l'Apgar a été estimé respectivement à 8/10 et 10/10 à la première et à la dixième minute. L'accouchement s'est compliqué d'une hémorragie de la délivrance jugulé par la perfusion d'ocytocique et un massage utérin. Le saignement s'est arrêté, mais l’état hémodynamique de la patiente s'altérerait, d'o[ugrave] son transfert dans notre formation.

A l'admission on trouvait une patiente obnubilé, polypnéique et tachycarde à 120 bat/min, la tension artérielle était à 70/40 mmHg. Après mise en condition notamment une oxygénothérapie et un remplissage par sérum salé, un examen général a pu être réalisé montrant une pâleur cutanéo-muqueuse généralisée. L'abdomen était légèrement distendu avec une sensibilité de l'hypochondre gauche. La rétraction utérine était satisfaisante sans saignement extériorisé. Une révision utérine sous couverture antibiotique a été réalisée faisant éliminer une rupture utérine. Un examen sous valve de la filière génitale était normal. Le bilan paraclinique objectivait une anémie à 4,5g/dl d'Hémoglobine nécessitant la transfusion de 6 culots globulaires. L’échographie abdominopelvienne révélait un épanchement de moyenne abondance, une rate augmentée de volume très hétérogène avec fracture splénique au niveau du pole supérieur, le foie et les reins étaient sans anomalies. Le balayage échographique sur l'utérus retrouvait une ligne de vacuité fine sans image de solution de continuité des parois utérines.

Une décision pluridisciplinaire d'une laparotomie d'urgence a été prise, l’équipe se composait de chirurgien obstétricien, un chirurgien viscéral et un réanimateur. Une incision médiane à cheval sur l'ombilic a été réalisée. [Agrave] l'ouverture on retrouvait un hémo-péritoine d'environ 3 litres de sang incoagulable avec des caillots. L'exploration du pelvis notait l'intégrité de l'utérus, des annexes et des paramètres. L'exploration de la loge splénique découvrait la rupture du pédicule vasculaire de la rate et une dilacération du parenchyme splénique avec suffusion hémorragique. Une splénectomie totale a été réalisée après ligatures des vaisseaux spléniques, suivie d'un drainage de la loge splénique et fermetures plan par plan. La patiente était stable sur le plan hémodynamique et fut transférée par la suite au service de réanimation o[ugrave] elle a était nouvellement transfusée.

Les suites opératoires étaient simples et les contrôles post-opératoires étaient normaux. La patiente est déclarée sortante dix jours après l'intervention.

## Discussion

La rupture de rate est une complication exceptionnelle de la grossesse et de l'accouchement. Habituellement elle est d'origine traumatique ou survient sur une rate pathologique (hémangiome, hémopathie, parasitose...). Il peut s'agir d'un traumatisme sous forme de contusions appuyées [1, 2]. D'autres événements de la vie peuvent être à l'origine d'une rupture de rate « spontanée » tels les vomissements, les éternuements, le coït et les efforts pour se mettre en position debout. Ces étiologies ont pour mécanisme une augmentation considérable de la pression intra-abdominale [3, 4].

La rupture peut aussi être causée par des traumatismes directs tels les blessures par armes blanches ou armes à feu [1]. Dans notre observation la rupture splénique faisait suite à des expressions abdominales responsables d'une augmentation de la pression abdominale. La pratique de ces expressions abdominales est d′usage courant mais sa fréquence réelle est inconnue [5].

Il n′existe aucune indication validée de l′expression abdominale, qui n′est “ni enseignée, ni codifiée, ni évaluée”. Cette manœuvre est souvent mal vécue par la patiente, induisant un stress physique et psychique immédiat et après l′accouchement, ainsi que des complications graves allant de simples douleurs abdominales persistantes, ecchymoses abdominales aux fractures de côtes, lésions périnéales, utérine ou déchirure du pédicule lombo-ovarien voire même une rupture hépatique ou splénique comme dans notre observation [5]. Les ruptures spléniques secondaires peuvent évoluer en deux temps, on observe d'abord une aggravation progressive avec des signes de choc et la constitution lente d'un hémopéritoine de volume croissant, ensuite survient le collapsus vasculaire par rupture de la collection intra- ou périsplénique [6]. L’échographie abdominale révèle un épanchement liquidien intra-abdominal, mais celui-ci peut être localisé dans la région périsplénique ou même absent si l’échographie est précoce. L'artériographie du tronc cœliaque permettrait d'affirmer la rupture de la rate, mais devant la gravité du tableau clinique, elle n'est pas réalisée et le plus souvent le diagnostic échographique est confirmé par la laparotomie. La ponction-lavage du péritoine, sous échoguidage, peut aider à orienter le diagnostic [7].

Le traitement repose initialement sur la réanimation cardiovasculaire, suivie en urgence d'une laparotomie afin de réaliser une splénectomie d'hémostase. L'abstention chirurgicale est possible à condition d'avoir un état hémodynamique stable avec épanchement minime [8].

Notre patiente avait présenté un état de choc avec instabilité hémodynamique, une anémie profonde nécessitant la transfusion de 6 culots globulaires et un épanchement d'abondance moyenne. Ces éléments cliniques étaient en faveur de la réalisation d'une splénectomie d'hémostase.

Le pronostic dépend de la précocité du diagnostic et de la prise en charge. Le diagnostic n'est évoqué que dans 25% des cas dans les contusions abdominales, et presque jamais dans le post-partum ce qui est responsable souvent d'une prise en charge retardée [3–9].

## Conclusion

La rupture de la rate peut compliquer des manœuvres manuelles telles les «expressions» abdominales, toujours pratiquées dans les formations sanitaires périphériques de certains pays en voie de développement. Le diagnostic est rendu difficile du fait de sa rareté et par la fréquence élevée des ruptures utérines qui font errer le diagnostic d'urgence. La prise en charge nécessite une réanimation et une splénectomie d'urgence. Le diagnostic et la prise en charge sans délai permettent d'améliorer le pronostic dans une pathologie o[ugrave] la mortalité reste élevée.
